# Genomic evidence of symbiotic adaptations in fungus-associated bacteria

**DOI:** 10.1016/j.isci.2025.112253

**Published:** 2025-03-20

**Authors:** Daniyal Gohar, Kadri Põldmaa, Mari Pent, Saleh Rahimlou, Klara Cerk, Duncan Y.K. Ng, Falk Hildebrand, Mo Bahram

**Affiliations:** 1 Institute of Ecology and Earth Sciences, University of Tartu, J. Liivi St. 2, 50409 Tartu, Estonia; 2 Department of Botany and Plant Pathology, Oregon State University, Corvallis, OR 97331, USA; 3 Natural History Museum and Botanical Garden, University of Tartu, Vanemuise 46, 51003 Tartu, Estonia; 4 Department of Ecology and Evolutionary Biology, University of Michigan, Ann Arbor, MI 48109, USA; 5 Gut Microbes & Health, Quadram Institute Bioscience, Norwich Research Park, Norwich, NR4 7UQ Norfolk, UK; 6 Earlham Institute, Norwich Research Park, Norwich, NR4 7UZ Norfolk, UK; 7 Department of Agroecology, Aarhus University, Forsøgsvej 1, 4200 Slagelse, Denmark; 8 Department of Ecology, Swedish University of Agricultural Sciences, Ulls väg 16, 756 51 Uppsala, Sweden

**Keywords:** Genomics, Microbial genomics, Microbial metabolism, Microbiology, Mycology

## Abstract

Fungi harbor diverse bacteria that engage in various relationships. While these relationships potentially influence fungal functioning, their underlying genetic mechanisms remain unexplored. Here, we aimed to elucidate the key genomic features of fungus-associated bacteria (FaB) by comparing 163 FaB genomes to 1,048 bacterial genomes from other hosts and habitats. Our analyses revealed several distinctive genomic features of FaB. We found that FaB are enriched in carbohydrate transport/metabolism- and motility-related genes, suggesting an adaptation for utilizing complex fungal carbon sources. They are also enriched in genes targeting fungal biomass, likely reflecting their role in recycling and rebuilding fungal structures. Additionally, FaB associated with plant-mutualistic fungi possess a wider array of carbon-acquisition enzymes specific to fungal and plant substrates compared to those residing with saprotrophic fungi. These unique genomic features highlight FaB’ potential as key players in fungal nutrient acquisition and decomposition, ultimately influencing plant-fungal symbiosis and ecosystem functioning.

## Highlights

• Fungus-associated bacteria have enriched carbohydrate metabolism and motility genes
• They may aid fungal metabolism through chitin- and trehalose-related genes
• EcM fungal bacteria have more plant- and fungal-substrate-acting genes than SAP
• Bacteria in fungal fruiting bodies lack nitrogen-fixing genes

## Graphical abstract

**Figure:**
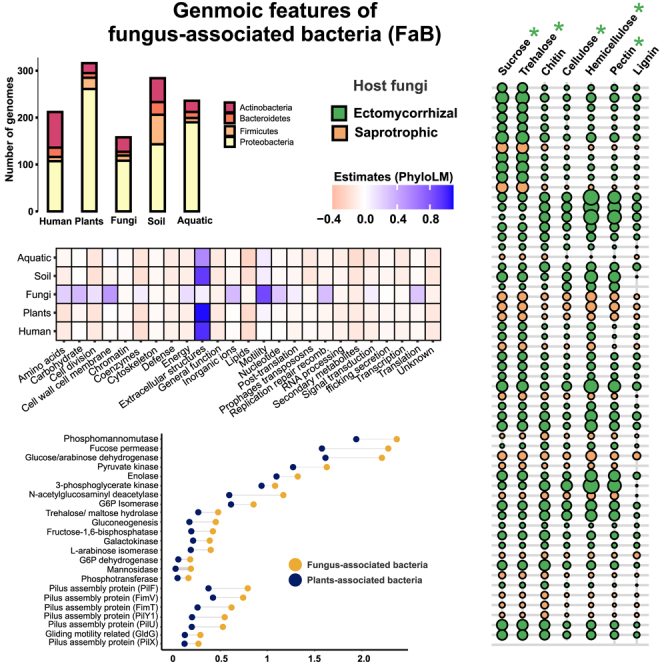

## Introduction

Bacteria, a major component of biodiversity on Earth,^1^ inhabit diverse environments, including organisms belonging to Eukaryota.^2^^,^^3^ Growing evidence suggests that bacteria influence the health of their hosts by promoting immunity, metabolism, and stress tolerance while also acting as pathogens that can negatively affect the host.^4^^,^^5^^,^^6^ Genomic studies of host-associated microbes point to their certain evolutionary adaptations. For example, plant symbiotic bacteria have expanded genomes compared to their free-living counterparts.^7^^,^^8^ At the same time, obligate symbionts of sap-feeding insects have smaller genomes and consequently encode only essential symbiotic proteins important for their adaptation.^9^ Several studies suggest that symbiotic bacteria may have lost some metabolic genes likely complemented by the host.^10^^,^^11^ Furthermore, free-living bacteria may have lost biosynthetic genes and gained genes related to metabolic cross-feeding interactions.^12^ Overall, bacterial genomes reflect their adaptation to host and habitat conditions.^13^^,^^14^ However, a comprehensive understanding of the genomic features of bacteria across various environmental settings and host-associated habitats remains elusive. Addressing this gap is crucial to illuminate the complex interplay between bacteria and their hosts, with implications for ecosystem functioning.

Among host-associated bacteria, the genomic features of those inhabiting fungal structures remain largely unexplored. Research has highlighted that fungus-associated bacteria (FaB) play a significant role in enhancing the fitness and resilience of fungi,^15^^,^^16^^,^^17^ promoting efficient substrate utilization,^18^^,^^19^ enhancing host growth,^20^^,^^21^ facilitating mycorrhizae formation,^22^^,^^23^ and inducing host sporulation.^6^^,^^24^ Moreover, FaB may confer protection against oxidative stress^25^^,^^26^ and may also be capable of nitrogen fixation.^27^^,^^28^ On the other hand, bacteria can also engage in antagonistic interactions, such as regulating the reproduction of their fungal hosts, with fungi-lacking endosymbiotic bacteria producing more spores than those inhabited by such.^29^ Furthermore, bacteria can inhibit fungal pathogenesis by producing antifungal compounds such as herbicolin.^30^ Several studies report that FaB have evolved certain genomic features to establish interactions with their host fungi. Examples include bacteria enhancing the pathogenicity^31^^,^^32^ and antibiotic and nutrient uptake ability of host fungi.^33^^,^^34^ Comparative phylogenomic studies of the fungi and their endosymbiont bacteria show that the presence or absence of endosymbionts highly modulates host metabolism.^11^ However, our understanding of the genomic features of FaB is still limited, particularly regarding their adaptation to their host fungi.

Here, we conducted a comparative genomics analysis on 56 newly sequenced and 107 FaB genomes in the public database, as well as available bacterial genomes from other habitats, including plants (*n* = 316), humans (*n* = 212), soil (*n* = 284), and water (*n* = 236). We hypothesized that FaB have evolved distinct genomic features compared to their counterparts in other habitats, enabling them to effectively colonize and thrive with their fungal host. Additionally, we hypothesized that FaB complement the nutrient acquisition strategies of their hosts, which should be reflected in contrasting genomic features of FaB in ectomycorrhizal (*n* = 39) and saprotrophic fungi (*n* = 17).

## Results and discussion

### Genome size and GC content

**Figure 1:**
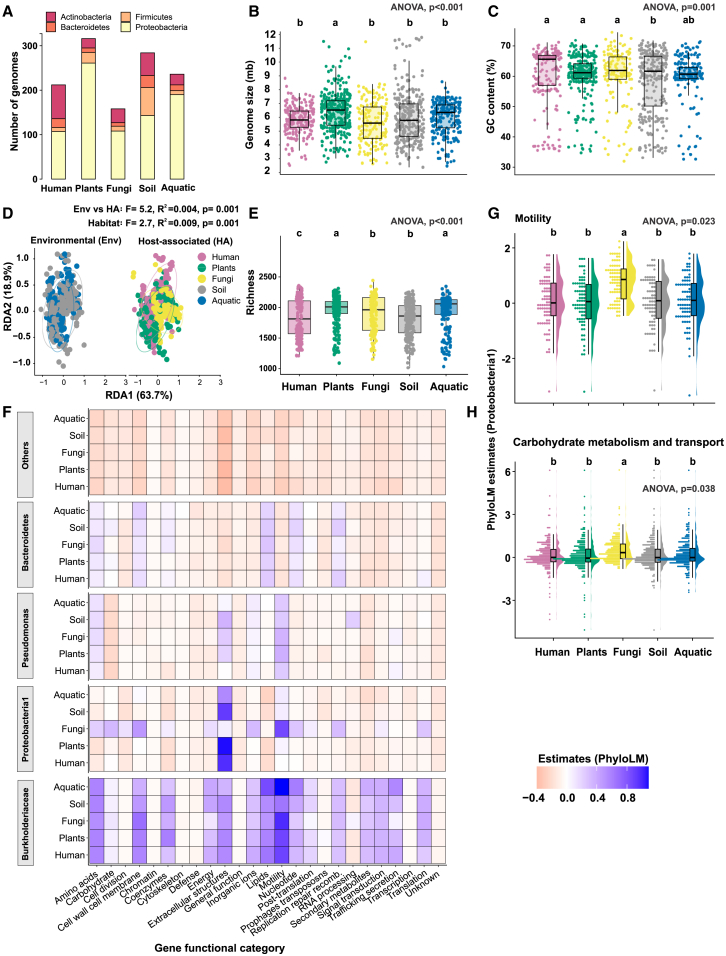
Diversity and enrichment patterns of genes in bacteria from various habitats (A) The number of genomes analyzed from each habitat. (B and C) The variations in bacterial genome size and GC content across different habitats. (D) The composition of functional gene orthologs (COGs) based on the Bray-Curtis distance matrix. (E) The richness of COGs from each habitat and (F) the differences in gene category enrichments across different habitats. The heatmaps indicate the level of enrichment or depletion, with blue and red cells representing overrepresentation and underrepresentation of gene categories, respectively, based on the PhyloLM estimates. The full COG category names for the x axis labels are in Table S2. Among the eight taxa, Proteobacteria 2, Bacillales, Alphaproteobacteria, and Actinobacteria were grouped under “Others” to improve the clarity of the figure. (G and H) The distribution of motility and carbohydrate metabolism and transport-related genes and their enrichment estimates are represented in alphaproteobacteria1, as shown in (F). Different letters above each boxplot indicate significant differences between groups. Similar letters show similarity between the compared groups, whereas different letters denote a statistically significant difference. ANOVA followed by *post hoc* tests (Tukey’s HSD) was used to test for differences between group pairs. All *p* values were corrected using the FDR method to control the false discovery rate.

To determine whether FaB exhibit expanded or reduced genomes compared to bacteria from other habitats, we compared the genome sizes of FaB against bacteria from plants, humans, soil, and water. Our results demonstrate a significant variation in genome size among the habitats studied, with the largest genomes observed for plant-associated bacteria (Figure 1B; Table S1) corroborating previous findings.^7^^,^^8^ The average genome size of FaB is comparable to that of soil bacteria, likely due to the predominant facultative symbiotic nature of FaB originating from soil.^35^ Although a common assumption is that symbiotic microbes have lost certain gene functions that are complemented by their hosts,^10^^,^^11^^,^^33^ plant-associated bacteria in our data exhibit larger genomes (Figure 1B), which may contribute to their diverse interactions with other microbes and their host plant.^36^^,^^37^^,^^38^ However, our data suggest that genome size reduction prevails in obligate endobacteria compared to environmental bacteria (soil and aquatic) that can also inhabit the surface of plants and fungal hyphae and most likely represent facultative symbionts. This is consistent with previous studies showing that the genomes of endohyphal facultative bacteria are more similar to their terrestrial counterparts, while not exhibiting reduction.^39^ However, our study revealed a similar distribution of GC content across bacteria inhabiting different hosts (humans, plants, and fungi) that is significantly higher than that observed in environmental bacteria (soil and aquatic; Figures 1C and S2B). Although GC content has been correlated with amino acid composition,^40^ nutrient availability,^41^ and growth temperature,^42^ it remains to be determined whether higher GC content in host-associated bacteria is functionally relevant.

### Bacterial functional diversity and composition reflect their habitat and host specificity

To compare the functional gene diversity and composition of bacteria across habitats, we examined their Pfam,^43^ COG,^44^ KEGG,^45^ and Tigrfam^46^ profiles using a redundancy analysis (RDA) of a Bray-Curtis distance matrix generated based on orthologous gene counts. The RDA plots reveal some degree of overlap in the clustering, which may reflect the complex and variable distribution of functional genes across the different habitats. Nevertheless, the statistical models indicate notable distinctions between the groups (Figures 1D and S3). These results suggest that the functional gene composition of bacteria can be explained by their habitat type (R^2^ = 0.07, *p* = 0.001) as well as host type (R^2^ = 0.06, *p* = 0.001) (Figure S3). The diversity of functional genes also significantly varied across habitats (Figures 1E and S4). The predominant role of bacterial habitat type in defining their functional gene diversity and composition may reflect the adaptive strategies of bacteria in response to the unique environmental conditions of their habitats.^47^ For example, fungi produce diverse secondary metabolites, which may impact the genomic and functional competency of bacteria striving to establish a symbiotic interaction with these fungi.^48^^,^^49^^,^^50^ Similarly, bacterial communities may respond to habitat-specific conditions, such as salinity, by activating or deactivating certain metabolic pathways,^51^^,^^52^ or they may be capable of transitioning between free-living and host-associated lifestyles, thereby evolving adaptive traits over time.^53^^,^^54^^,^^55^ Our results suggest that bacterial functional diversity and composition reflect their habitat and host specificity.

### FaB are enriched in carbohydrate metabolism and cell motility genes

To test the hypothesis that FaB may have evolved specific genomic adaptations to colonize and thrive in their fungal habitat effectively, we performed the PhyloLM test.^56^ We evaluated gene enrichment patterns in FaB genomes compared to genomes of bacteria from other habitats. PhyloLM accounts for phylogenetic relationships, thereby eliminating enrichments that may result from shared ancestry, resulting in an accurate assessment of gene enrichments in the given groups.^8^^,^^56^ Although we accounted for phylogeny in our analysis, a key limitation was the unequal representation of certain taxa from different habitats, due to the exclusion of genomes for which required data were missing. For instance, Pseudomonadales were more prevalent in aquatic habitats due to a higher number of sequenced genomes from these environments. While our primary goal was to compare FaB with bacteria from other habitats, we included a maximum number of high-quality genomes of similar taxa sequenced from different habitats. Nonetheless, based on our data, the following gene categories were consistently enriched regardless of bacterial phylogeny and habitat type: *amino acids, lipids, replication and recombination*, *secondary metabolites, and signal transduction and translation* (Figure 1F). Compared to other habitats, the genes related to carbohydrate *transport and metabolism*, *cell wall and cell membrane*, and *cell motility* were significantly more enriched in the genomes of a Proteobacteria cluster (hereafter Proteobacteria1) represented among FaB (Figures 1G and 1H). These genes may enable FaB to efficiently colonize surfaces of fungal cells that receive carbon compounds through symbiotic association with plants or by saprotrophic means and to travel along the hyphae. Moreover, bacteria may employ these genes to locate fungal surfaces and respond to fungal exudates in their surrounding environment. These results provide evidence for the importance of carbohydrate transport and metabolism genes in facilitating bacterial colonization of FaB.

**Figure 2:**
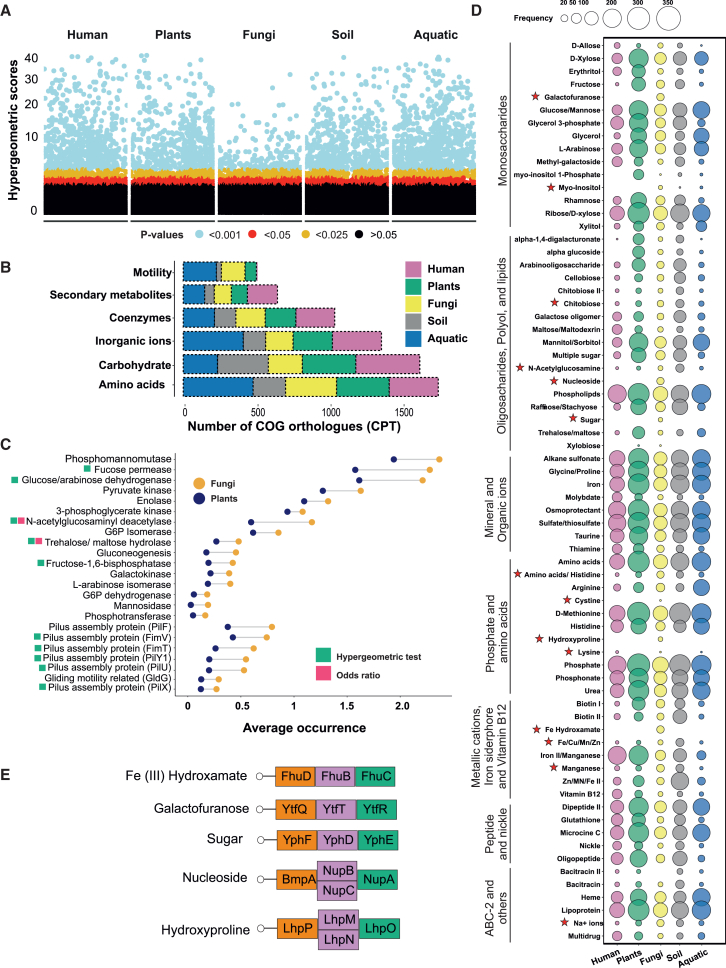
Comprehensive analysis of FaB genes (A) The analysis of association of gene orthologs (COGs) with their respective habitat was performed using hypergeometric tests. The y axis represents the hypergeometric scores, while the color-coded points signify *p* values indicating a significant gene association with a particular habitat. (B) The functional categories of habitat-associated genes and the number of genes (COGs) significantly associated with a specific habitat from each functional category. We transformed absolute gene numbers into the gene counts per thousand genomes (CPTs) to account for the varying number of genomes in each habitat. For better visualization, only the most abundant gene functional categories are illustrated (general function and unknown function were omitted). However, a list of the genes associated with habitats can be found in Table S3. (C) Significantly overrepresented genes (FDR-corrected *p* value < 0.05; Table S4) in FaB compared to plant-associated bacteria. The x axis represents the average occurrence of each gene on the y axis in fungi- and plant-associated bacteria. The color codes on the y-axis labels represent the statistical validation of the association of genes with FaB. To simplify the illustration, we only included genome data of bacteria from plants and fungi, since both of these host-associated habitats occupy a similar niche, in contrast to the habitats in humans. (D) Comparison of ABC transporter pathways in bacterial genomes across different habitats. Only the complete pathways (with all genes present in a genome) were counted to determine the frequency of complete pathways in genomes from each habitat. The list of genes involved in each pathway is provided in Table S6. The red stars with the pathway name represent where FaB distinguishes itself from other habitats or has more pathway copy numbers than plant-associated bacteria. Frequency of pathway copy number was compared using chi-squared tests followed by *p* value correction using the FDR method to control for false discovery rate. (E) Illustrate the list of the pathways and the genes involved in each pathway that were were found to be specific to FaB, compared to bacteria from other habitats.

To further investigate the genes involved in bacterial adaptation to various habitats, we used a hypergeometric test that considers factors such as sample size and gene frequency to identify genes associated with each habitat (Figure 2B). Despite observing some overlap in gene associations among habitat types (Figure S5), the principal coordinate analysis revealed distinct clusters that separated FaB genes from those originating from other habitats, supporting our results from PhyloLM tests (Figure S6). Among broad gene categories, FaB genes mainly belonged to *Unknown function* or *General function*, while *Carbohydrate metabolism and transport and Motility-related genes* were also observed (Table S3). To further validate these results, we compared overrepresentation patterns of all genes related to *carbohydrate metabolism* and *cell motility* (Figure 1F). We observed 23 genes significantly overrepresented (Figure 2C) in fungi but depleted in other habitats. Among these, 10 genes were identified as FaB-specific genes, as determined by either hypergeometric or odds ratio tests, including various genes for carbohydrate metabolism and bacterial pilus assembly required for motility. These findings provide evidence for the importance of carbohydrate metabolism and bacterial pilus assembly-related genes for bacterial adaptation to fungal habitats (the complete list of FaB-specific genes is provided in Table S3).

### Contribution of FaB to fungal metabolism

**Figure 3:**
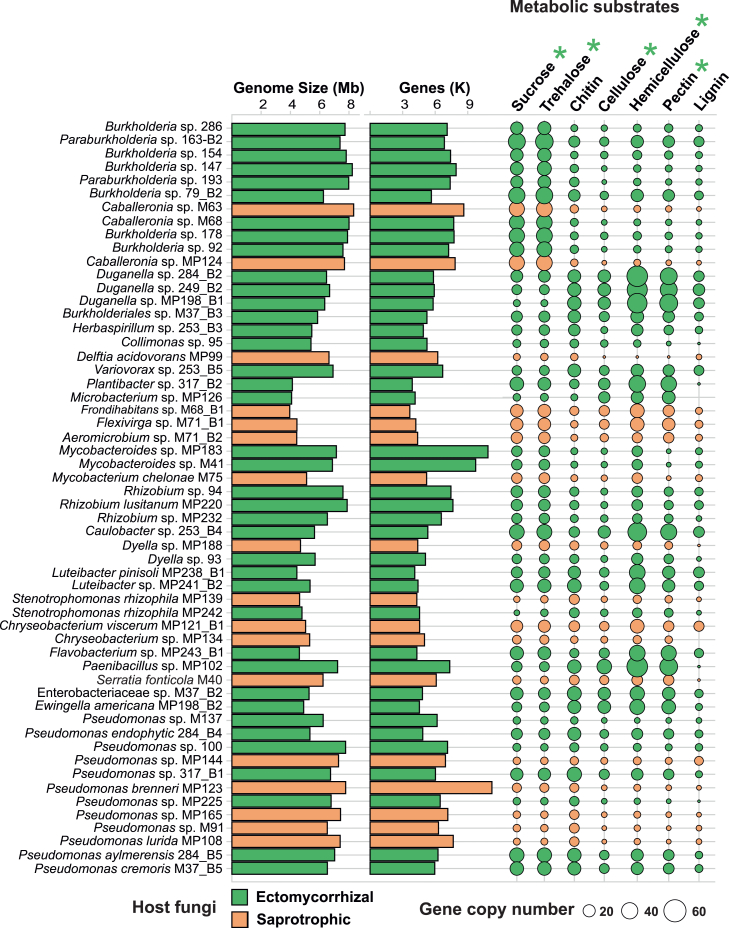
FaB CAZyme repertoires acting on fungal and plant-derived substrates The number of metabolic enzymes acting on fungal and plant substrates is represented by the point size. The different lifestyles of host fungi (e.g., ectomycorrhizal and saprotrophic) are color-coded. A complete list of CAZyme families involved in the metabolism of each substrate can be found in Table S5. A star symbol indicates that a metabolic substrate is significantly more abundant in ectomycorrhizal than saprotrophic fungi (*p* < 0.03, FDR corrected).

Our analysis did not uncover the evidence of nitrogen fixation (nifHDK genes) by FaB, despite previous speculations about their potential nitrogen-fixing abilities.^28^^,^^57^^,^^58^ However, we found that FaB can potentially influence fungal growth and biomass production, which is exerted through their impact on the biosynthesis and degradation of trehalose and chitin within the fungal cell wall. This is evidenced by the overrepresentation and significant association of genes coding for enzymes related to trehalose and chitin metabolism with FaB (FDR corrected *p* < 0.05; Figures 2C and 3). Chitin and trehalose are pivotal constituents of fungal biomass, wherein the fungal cell wall is predominantly made up of chitin, an N-acetylglucosamine-based polymer, and trehalose, a disaccharide, serves as a carbohydrate repository that has structural and functional roles.^59^^,^^60^ Compared to plant-associated bacteria, the higher abundance of N-acetylglucosaminyl deacetylase (NAGd) genes (Figures 2A and 2C; FDR corrected *p* < 0.01; Figure 3; Table S4), responsible for chitin degradation,^61^ in FaB may reflect the fungal need for chitin-active enzymes during developmental processes such as mycorrhizae or fruiting body formation.^62^^,^^63^ Hydrolysis of chitin releases monomers or oligomers that can be used as carbon and nitrogen sources by both bacteria and fungi.^64^ Alternatively, chitin-degrading capabilities may enable bacteria to target or degrade their fungal hosts. The presence of NAGd enzymes in bacteria could also enhance ability of fungi to scavenge nitrogen from organic matter, a key nitrogen source for mycorrhizal fungi.^65^^,^^66^^,^^67^ Recent research has shown that a significant portion of nitrogen, which would otherwise be unavailable, can be utilized by mycorrhizal fungi in the presence of *Paenibacillus* sp.^68^ These observations suggest that, under certain conditions, FaB may enhance the nutrition of their host fungi. However, such interactions would require further investigation.

Through their role in trehalose metabolism (Figures 2A, 2C, and 3), FaB may boost their hosts’ stress tolerance, growth and spore germination. Trehalose is synthesized and stored in fungal hyphae^69^^,^^70^ and is utilized as an energy source for spore germination and fruiting body development.^71^^,^^72^^,^^73^ Trehalose has been shown to accumulate in the cell wall during certain stages of fungal growth. It may affect the structural properties of the cell wall, potentially contributing to the strength and resilience of the fungal hyphae.^59^^,^^72^^,^^73^^,^^74^^,^^75^ Furthermore, trehalose secreted by plant-associated microorganisms can act as a sucrose analog, influencing host growth by regulating carbon allocation and potentially influencing assimilate allocation in surrounding cells or tissues.^76^^,^^77^ Our analysis of carbohydrate-active enzyme profiles revealed that FaB possess a set of CAZymes, namely GT4, GT20, and GH37, necessary for trehalose synthesis and hydrolysis^78^ (Figure 3; Table S5). These findings suggest that these enzymes may be involved in trehalose metabolism, potentially contributing to the growth, stress tolerance, and biomass production of fungi. The presence of GT4, GT20, and GH37 implies that FaB possesses the necessary enzymatic machinery to regulate trehalose levels, a process that may be critical for its metabolic flexibility, energy storage, and dynamic response to environmental stress.

Other genes that were overrepresented in FaB encode enzymes involved in the metabolism of oligo- and polysaccharides (Figure 2C; FDR-corrected *p* < 0.05; Table S4), including galactose (Galactokinase; EC 2.7.1.6) and glucose (glucose dehydrogenase), as well as of transporters and hydrolases (Table S4). Furthermore, seven of these genes encode enzymes fundamental to glycolysis in bacteria, including enolase (EC 4.2.1.11), glucose dehydrogenase (EC 1.1.1.47), pyruvate kinase (EC 2.7.1.40), and phosphoglycerate kinase (EC 2.7.2.3). The abundance of simple sugars like glucose in fungal habitats suggests that glycolysis plays a crucial role in FaB metabolism, allowing for direct energy production from readily available nutrients.

### The high dispersal ability of FaB

Pilus assembly is critical for bacterial adhesion to surfaces and host-bacterial interactions.^79^^,^^80^ This mechanism is a significant determinant of bacterial virulence, movement along surfaces, and colonization in host organisms.^81^^,^^82^^,^^83^ Enrichment of genes related to motility, such as pilus assembly in FaB (Figures 1G and 2C), suggests that these features may allow bacteria to reach areas that would otherwise be inaccessible and to travel along the surface of fungal hyphae. Fungal hyphae can create a relatively more aqueous environment due to water films around and within the hyphal network^84^^,^^85^ compared to the surrounding environment. Bacteria potentially move more easily on hyphae due to the water films, which reduce friction and provide a pathway for bacterial movement.^81^^,^^82^^,^^86^ This is in line with a previous study showing that mycorrhizal networks facilitate the colonization of legume roots by symbiotic nitrogen-fixing bacteria.^87^ Our results provide genomic evidence that cell mobility-related genes may play a crucial role in bacterial adaptation to fungi and that the dominant mode of bacterial movement on fungal highways is facilitated through pili.

### Nutrient acquisition capability of FaB

We further found that FaB possess diverse nutrient acquisition capabilities as reflected by the presence of an array of ATP-binding cassette (ABC) transporter pathways (Figure 2D; Table S6), which are responsible for the uptake and transport of carbohydrates, minerals, amino acids, and vitamins. In particular, compared to bacteria associated with other environments, FaB exhibited a significantly higher abundance of transporter pathways for certain essential carbohydrates, amino acids and ions, such as galactofuranose and Fe hydroxamate (FDR corrected, chi-squared *p* < 0.05; Figures 2D and 2E), which suggests that FaB possess a distinctive ability to obtain nutrients from its surroundings. Furthermore, FaB likely use galactofuranose, a key component of fungal hyphae,^88^ as a means of lipopolysaccharide communication,^89^^,^^90^ to recognize, infect, and colonize their fungal host, as reflected in the exclusive presence of pathways encoding proteins responsible for galactofuranose uptake in FaB (Figure 2D; Table S6). Similar interactions involving bacterial attachment to fungal hyphae through lipopolysaccharides have been reported for *Agaricus bisporus*^91^ and arbuscular mycorrhizal fungi,^92^ and the use of polysaccharides by bacteria has been shown to enhance their ability to evade recognition by the host immune system.^88^ Furthermore, the transporters may facilitate the exchange of nutrients between partners and optimize resource utilization in symbiosis. For instance, bacterial Fe (III) hydroxamates, iron-chelating compounds,^93^ may contribute to iron transport by fungi to their plant hosts.^94^^,^^95^^,^^96^

### FaB may complement the functions of their hosts

Our analysis further revealed contrasting genomic features of FaB depending on the nutritional modes of their host fungi (Figure 3), suggesting a potential role of FaB in the functioning of their host fungi. Specifically, we found that metabolic enzymes acting on certain plant substrates, including sucrose, cellulose, and hemicellulose, were significantly more abundant in ectomycorrhizal fungi-associated bacteria than in bacteria associated with saprotrophic fungi (Figure 3; Table S5). This contrasts comparative genomics studies of fungi reporting fewer CAZymes for polysaccharides in ectomycorrhizal fungal genomes compared to saprotrophic fungi.^97^ Ectomycorrhizal fungi are usually adapted to environments with low litter quality and thus need extensive enzymes for obtaining nutrients from organic matter.^98^^,^^99^ It is, therefore, tempting to speculate that a greater C-degradation potential of bacteria may allow ectomycorrhizal fungi to develop diverse nutrient acquisition strategies when the supply from plant hosts suffers.

To investigate the genomic potential of FaB to degrade sucrose, which may play a role in fungal-plant interactions, we performed an *in vitro* experiment. Specifically, we cultivated three analyzed bacterial strains, irrespective of the guild of their associated fungi, with genes for GH32 enzymes (encoding for Invertase: EC 3.2.1.26) on media containing sucrose as the carbon source. We focused on sucrose as it is the primary source of C for mycorrhizal fungi in the apoplast of the root-fungal interface.^100^^,^^101^^,^^102^ Our results confirmed the capacity of tested FaB strains (in two out of three) to utilize sucrose as a carbon source (Figure S8). Unlike saprotrophic fungi, ectomycorrhizal fungi lack sucrose invertases^103^^,^^104^^,^^105^ and rely on plant cell wall-bound invertases to obtain digestible monosaccharides.^101^^,^^106^ By regulating the activity of sucrose invertases, plants can modulate the allocation of sugars for fungi, influencing fungal growth.^100^^,^^107^^,^^108^^,^^109^ However, our analysis of CAZyme repertoires (Figure 3) suggests that most FaB have the potential to play a key role in the inversion of sucrose, augmenting host functions and contributing to the gradient of sucrose concentration at the root-fungal interface. Previous studies have reported the observation of similar complementary behavior of bacteria in tripartite interactions of plants, fungi, and bacteria.^15^^,^^110^ For example, FaB enhance their host fungi’s responsiveness to strigolactones exuded by roots, leading to increased hyphal elongation and branching relevant to mycorrhizal establishment.^21^^,^^111^ Our findings, therefore, underscore the potential of FaB in metabolizing sucrose and support that FaB symbiotically complement their fungal hosts in the degradation and utilization of carbohydrates.

### Conclusions

This study identified distinctive genomic features that may enhance the colonization of fungi by bacteria and uncovered the potential key role of carbohydrate metabolism and cell mobility in bacterial-fungal interactions. Moreover, our findings suggest that FaB have evolved more specialized adaptations for interacting with mycorrhizal fungi than saprotrophic fungi and that FaB may exploit fungal hyphae for nutrient acquisition and interacting with plants. These findings underscore the importance of genomic adaptations in facilitating bacterial-fungal interactions and point to the important role of FaB in enhancing ecosystem functioning through their support to host fungi.

### Limitations of the study

The shortcomings of this study stem from the number of available FaB genomes and the limited range of environments analyzed. Another limitation was the incomplete metadata for genomes retrieved from public databases, particularly the lack of host taxonomy and geographical information. However, for the analysis of CAZyme associated with ectomycorrhizal-versus saprotrophic fungi, we focused on the newly generated genomes, which helped to reduce biases that could be caused by geographical variation and ensured that genomes with only well-characterized host associations were included. Finally, while this study provides evidence for the distinct genomic potential of FaB, their realized functions remain to be examined in future studies.

## Resource availability

### Lead contact

Any further information or requests for resources and methods should be directed to the lead contact, Daniyal Gohar (gohard@oregonstate.edu).

### Materials availability

The isolates analyzed in this study are preserved in the microbiological collection of the Natural History Museum and the Collection of Environmental and Laboratory Microbial Strains (CELMS) at the University of Tartu. The isolates can be made available upon request to the collections’ curators.

### Data and code availability

• The data supporting the results of this study are available in the public database JGI/IMG, with accession numbers provided in Table S1. Alternatively, the data can be accessed as separate datasets at https://doi.org/10.5281/zenodo.10889199.
  Dataset 1: Nucleotide FASTA files (assembled).
  Dataset 2: Annotations.
  Dataset 3: CAZyme annotations.
  Dataset 4: Metadata.
• The script used for this study’s bioinformatics and statistical analyses can be accessed at https://github.com/daniyalgohar/Genomic-features-of-FaB.

## Acknowledgments

We thank Jessie Uehling and Age Brauer for their constructive comments on an earlier version of the manuscript. M.B. was supported by the Swedish Research Council Vetenskapsrådet (grant 2021–03724) and the 10.13039/501100009708Novo Nordisk Foundation (NNF). F.H. was supported by the European Research Council H2020 StG (erc-stg-948219, EPYC), and D.Y.K.N. and F.H. by H2020-EU.3.2.2.3. (grant no. 863059 – www.fns-cloud.eu). K.C., D.Y.K.N., and F.H. were supported by the 10.13039/501100000268Biotechnology and Biological Sciences Research Council Institute Strategic Program Food Microbiome and Health BB/X011054/1 and its constituent project BBS/E/F/000PR13631 as well as Decoding Biodiversity BB/X011089/1 and its constituent project(s) BBS/E/T/000PR13638.

This work was supported by the 10.13039/501100002301Estonian Research Council grant PRG1170 and the European Union through the European Regional Development Fund (the Centers of Excellence: EcolChange and AgroCropFuture), Swedish Research Council Vetenskapsrådet (grant 2021–03724), and 10.13039/501100001862Formas (grant 2020–00807).

## Author contributions

D.G., K.P., and M.B. set up and designed the study. D.G. performed the most of the data analysis and wrote the manuscript. M.P. conducted bacterial culturing, DNA extraction, and library preparation for sequencing. S.R. contributed to quality filtering and genome assembly. K.C., D.Y.K.N., and F.H. helped with bioinformatics and statistical analysis. M.B. and K.P. supervised the study. D.G. wrote the first version of the manuscript with input from M.B. All authors contributed to the manuscript revision and approved the submitted version.

## Declaration of interests

The authors declare no competing interests.

## STAR★Methods

### Key resources table

**Table:** 

REAGENT or RESOURCE	SOURCE	IDENTIFIER
**Deposited data**		

Genome data of 56 fruiting body associated bacterial Isolates	This study	https://doi.org/10.5281/zenodo.10889199

**Software and algorithms**		

DOE-JGI Microbial Genome Annotation Pipeline (MGAP)	Chen et al.^112^	https://img.jgi.doe.gov/docs/pipelineV5/
CheckM	Parks et al.^113^	https://doi.org/10.7287/peerj.preprints.554v1
Trimmomtic	Bolger et al.^114^	http://www.usadellab.org/cms/?page=trimmomatic
Megahit	Li et al.^115^	https://github.com/voutcn/megahit
pyANI	Pritchard et al.^116^	https://pyani.readthedocs.io/en/latest/about.html
UBCG pipeline	Na et al.^113^	https://help.ezbiocloud.net/ubcg-users-manual/
MAFFT	Katoh et al.^117^	https://mafft.cbrc.jp/alignment/software/
FastTree	Price et al.^118^	https://morgannprice.github.io/fasttree/
ape	Paradis et al.^119^	https://cran.r-project.org/web/packages/ape/index.html
hclust	Tibshirani et al.^120^	https://www.rdocumentation.org/packages/stats/versions/3.6.2/topics/hclust
RPS-blast	Lu et al.^121^	https://www.ncbi.nlm.nih.gov/Structure/cdd/cdd_help.shtml#RPSBWhat
pfam database	Mistry et al.^43^	http://pfam.xfam.org/
COG database	Tatusov et al.^44^	https://www.ncbi.nlm.nih.gov/research/cog
KEGG Pathway database	Kanehisa and Goto^45^	https://www.genome.jp/kegg/
Tigrfam	Haft et al.^46^	https://tigrfams.jcvi.org/cgi-bin/index.cgi
dbCAN pipeline	Yin et al.^122^	https://github.com/linnabrown/run_dbcan

### Method details

#### Study sites and fungal sampling

Fungal fruiting bodies from different genera were collected from boreal forests at three nature reserves in Eastern Estonia. Only mature fruiting bodies were sampled, excluding the decayed or damaged fruiting bodies. All fruiting bodies were individually packed in aluminum foil, transported to the lab using a cold container and stored at 4°C until processed in a laminar hood.

#### Culturing, DNA extraction and library preparation

Fruiting bodies underwent careful cutting and sterilization procedures. Subsequently, 5 mm^3^ inner tissue sections of the fruiting bodies were used to liberate bacteria from these. Detailed methodologies pertaining to sample handling and bacterial culturing are described in Pent et al., 2017.^35^

After pure cultures of bacteria were established, their colonies were subjected to DNA extraction, followed by PCR amplification of 16S gene for identification, as outlined in Pent et al., 2017.^35^ The isolated strains were preserved at -80°C in 50% glycerol solution in the microbiological collection of the Natural History Museum and the Collection of Environmental and Laboratory Microbial Strains (CELMS) at the University of Tartu.

Based on the 16S rDNA-based identification of bacterial strains, we selected the 56 potential nitrogen-fixing bacteria for whole-genome sequencing. The sequencing library for each DNA extract was prepared using the NEXTRA XT kit, which fragments DNA and tags it with sequencing adapters in a single-tube enzymatic reaction.

#### Sequencing

DNA from libraries was sequenced on a next-generation sequencing platform using Illumina Novoseq 2xPE150 technology at Novogen corporation Inc. (U.K). Sequenced DNA was quality-filtered and assembled *de novo* via Trimmomtic^114^ and Megahit,^115^ respectively. The assembly quality was assessed using BUSCO v3 and CheckM with default parameters. Scaffolds smaller than 500 bp were removed. Assembled genomes were further annotated using the DOE-JGI Microbial Genome Annotation Pipeline (MGAP).

#### Genome data selection and filtration

We retrieved 4,388 bacterial genomes (belonging to similar genera or to the lowest taxonomic rank in common to strains included from our collection) inhabiting different habitats such as the human body, soil, fungi (mycelia, fruiting-bodies, mycorrhizal root tips), plants (roots, leaves, stems, branches), and water bodies (oceans, lakes) from the IMG database.^112^ Isolation sites were identified through manual curation, including scanning IMG metadata and NCBI biosample data and literature where required. Bacteria were classified as either host-associated or environmental bacteria, based on the available information on the host, habitat type or isolation source. Bacteria isolated from structures of humans, plants, and fungi were classified as host-associated bacteria regardless of the nature of their interactions with their host. Similarly, those isolated from terrestrial and aquatic habitats were classified as environmental bacteria. We found 138 genomes of fungus-associated bacteria from the public database. Following the quality filtering, only 107 genomes were included in the subsequent analysis, 83 taxonomically corresponding to newly generated genomes while others were included to increase the sample size for FaB. While metadata for the source of host fungi was missing for many FaB genomes, the majority of FaB included in this study originate from either fungal fruiting bodies or the mycelia in the soil, as indicated by the metadata in the public database. However, for a comprehensive comparison of fungus-associated bacteria, we added 56 newly sequenced genomes from fruiting bodies representing different fungal lineages, bringing the total number of FaB genomes to 163 (Table S1).

We applied the following rigorous quality control measures to ensure the selection of a quality and unbiased set of genomes in the subsequent analysis: (1) Based on the metadata and taxonomy, we selected up to 5 random genomes of the same species from the same habitat; (2) Genomes with missing or ambiguous isolation sites were discarded; (3) we used CheckM^113^ to assess the genome completeness and contamination, genomes that were predicted to be less than 95% complete and have more than 5% contamination were removed. In addition, we removed the genomes that did not have at least 90% of the 92 single-copy orthologous genes^9^; (4) To evaluate high-quality annotation, genomes with less than 85% protein-coding sequences were discarded; and (5) We used pyANI^116^ to compute the average nucleotide identity and alignment fraction values for each pair of genomes. We marked the genome pair as redundant when the alignment fraction exceeded 90%, and the average nucleotide identity was greater than 99.995%. In such a case, one genome was randomly selected, and the other was filtered out. This led to the exclusion of many obligate and endosymbiotic bacteria due to their smaller genome sizes, which did not meet our quality filtering criteria. However, we chose to apply the same criteria uniformly across all habitats, even though this resulted in the exclusion of some data. Finally, we selected 1211 genomes from four different phyla, as shown in Figure 1A, for the final analysis.

#### Construction of the phylogenetic tree

To generate a phylogenetic tree of 1211 high-quality non-redundant genomes, we retrieved 92 single-copy homologous genes from each genome with a UBCG pipeline.^123^ Each marker gene codon-based alignment (amino acid sequences) was aligned using MAFFT^117^ with default parameters. All 92 gene alignments were then concatenated into an overall merged alignment, which was used to build a maximum-likelihood phylogenetic tree with FastTree.^118^

#### Clustering of 1211 genomes into eight clusters

We divided 1211 genomes into eight taxa to allow downstream gene enrichment analysis. We first converted the phylogenetic tree into a distance matrix using a cophenetic function from the “ape” package in R.^119^ We then clustered 1211 genomes into eight clusters using the hierarchical clustering method using the function “hclust”. The algorithm clusters *n* objects based on pre-defined k-mer numbers. To determine the optimal value of *k* we used silhouette and gap statistics.^120^ We selected *k*=8 because it yielded a maximum silhouette coefficient (0.77) (Figure S1). The resulting clusters have few overlapping genomes that might have appeared because of false taxonomic identification provided with their metadata. Our clustering divided the Proteobacteria genomes into two taxa that we renamed, for simplicity, Proteobacteria1 and Proteobacteria2.

#### Gene category enrichment analysis

We retrieved protein-coding genes, for each genome using the program RPS-blast^121^ at an e-value cutoff of 1e-4 and alignment length of at least 70% of the consensus sequence length, and mapped them to COG (Cluster of Orthologous) IDs. Each COG ID was translated to at least one COG category (Table S2). We then counted the number of genes from a given category for individual genomes. We then assessed the enrichment patterns of gene categories across different habitats using Phylogenetic linear Regression (PhyloLM) and Hypergeometric tests. PhyloLM incorporates phylogenetic information to account for enrichments that may arise due to shared ancestry,^8^^,^^49^ while the hypergeometric test focuses solely on statistical enrichment based on categorical data without considering evolutionary relationships.^124^ Both methods were used to validate and complement the identified enrichment patterns, ensuring robust results. Furthermore, to reduce the bias, we used standardized gene copy numbers as input and predicted the effect size for individual genes. Additionally, we utilized the median of genes for each category to present our results, providing a comprehensive analysis of gene enrichments across the habitats.

#### ABC transporters

To identify the ABC transporter pathways within bacterial genomes across habitats, we sourced information on transporter genes associated with each pathway from the Kyoto Encyclopedia of Genes and Genomes (KEGG) database.^45^ We specifically tallied complete pathways, which contain all necessary genes within a genome, to assess the prevalence of complete pathways in bacterial genomes from different habitats. The list of genes involved in each pathway is presented in Table S6.

#### Genome-wide analysis of CAZyme domains

To analyze the genome-wide carbohydrate-active enzyme (CAZyme) related genes, we used the dbCAN pipeline.^122^ The protein sequences were annotated using DIAMOND, HMMER, and eCAMI algorithms integrated within dbCAN against CAZy and dbCAN databases. To avoid ambiguous annotations, we filtered out the genes not annotated by at least two algorithms. We then used custom R scripts that included basic data manipulation of results obtained from the dbCAN. First, we extracted all the CAZyme domains present in each genome collated them by domain family, and then summarized them into a table including the count of all the CAZyme domains in each family per genome. These collated and counted values of domain frequency per CAZyme domain family per genome were visualized using R.

### Quantification and statistical analysis

Unless otherwise mentioned, we used R, using the latest available version at the time of analysis, to statistically analyze the data. We distinguished functional repertoires between habitat types using distance-based redundancy analyses (dbRDA) and permutational multivariate analysis of variance (PERMANOVA) based on Bray-Curtis dissimilarity indices between genomes calculated from gene copy numbers represented in The Protein families (Pfam), COG, KEGG, and The Institute for Genomic Research's database of Protein Families (Tigrfam) annotations (host-associated and environmental). Effects of estimates of PhyloLM models were predicted using the “Predict” function, and median values of predicted residuals for each category and habitat were compared using pairwise comparison tests. All of the pairwise comparisons in this study were performed using Wilcoxon tests and t-tests, Kruskal-Wallis, and Mann-Whitney U test. P-values resulting from all tests were corrected using the False Discovery Rate (FDR) method. Furthermore, the overrepresentation of genes was also compared using pairwise tests. Hypergeometric and Odds Ratio tests were used to test the association of genes with their respective habitat. The Odds Ratio test is a statistical method used to measure the strength of association between two variables by comparing their probability of occurrence in different groups.^125^

## Supplemental information

Document S1. Figures S1–S8

Table S1. Metadata of the genomes used in the analysis

Table S2. Full COG category names used in Figure 1F

Table S3. Results of hypergeometric tests (Figure 2B)

Table S4. List of genes from the “Carbohydrate metabolism and transport” category that are more abundant in fungal-associated compared to plant-associated bacterial genomes (refer to Figure 2C)

Table S5. List of plant and fungal substrates along with their corresponding acting enzymes (refer to Figure 3)

Table S6. List of genes involved in ABC transporter pathways analyzed (refer to Figure 2D)
