# Supplementary material for: Genomic evidence of symbiotic adaptations in fungus-associated bacteria
Source: iScience. 2025 Mar 20;28(4):112253. doi: 10.1016/j.isci.2025.112253 (PMC12023794; doi:10.1016/j.isci.2025.112253)
Supplement: Document S1. Figures S1–S8 [file mmc1.pdf]

iScience, Volume *28*

Supplemental information

Genomic evidence of symbiotic adaptations in fungus-associated bacteria

Daniyal Gohar, Kadri Põldmaa, Mari Pent, Saleh Rahimlou, Klara Cerk, Duncan Y.K. Ng, Falk Hildebrand, and Mo Bahram

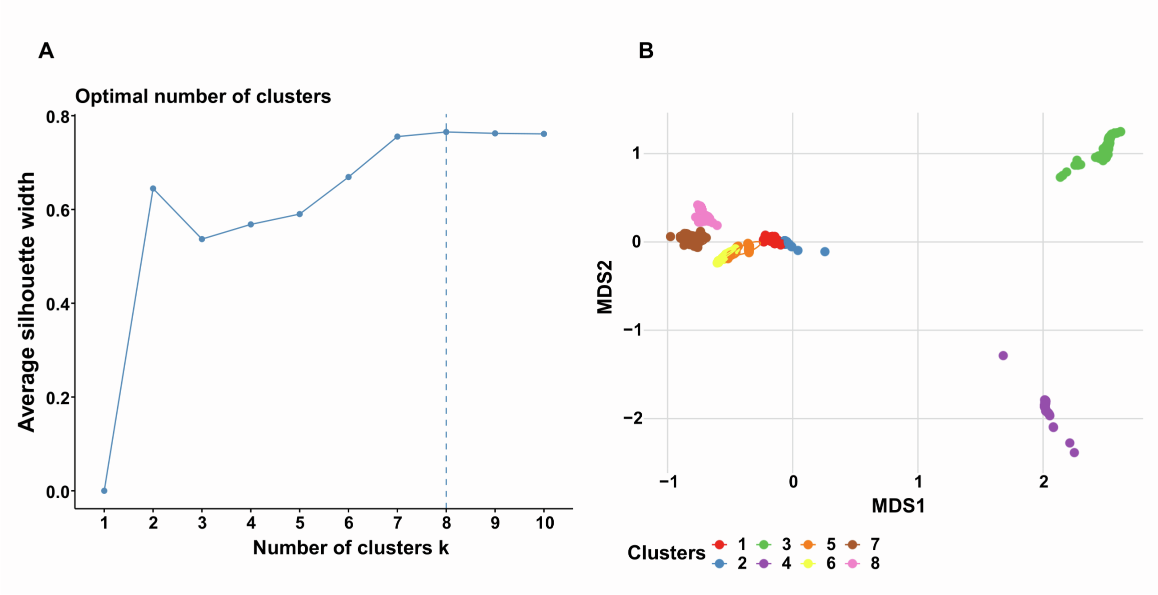

Figure S1: **Hierarchical clustering of 1211 genomes into 8 phylogenetically related taxa.** The dotted vertical line shows the K value with maximum silhouette width coefficient, which was selected for further analysis.

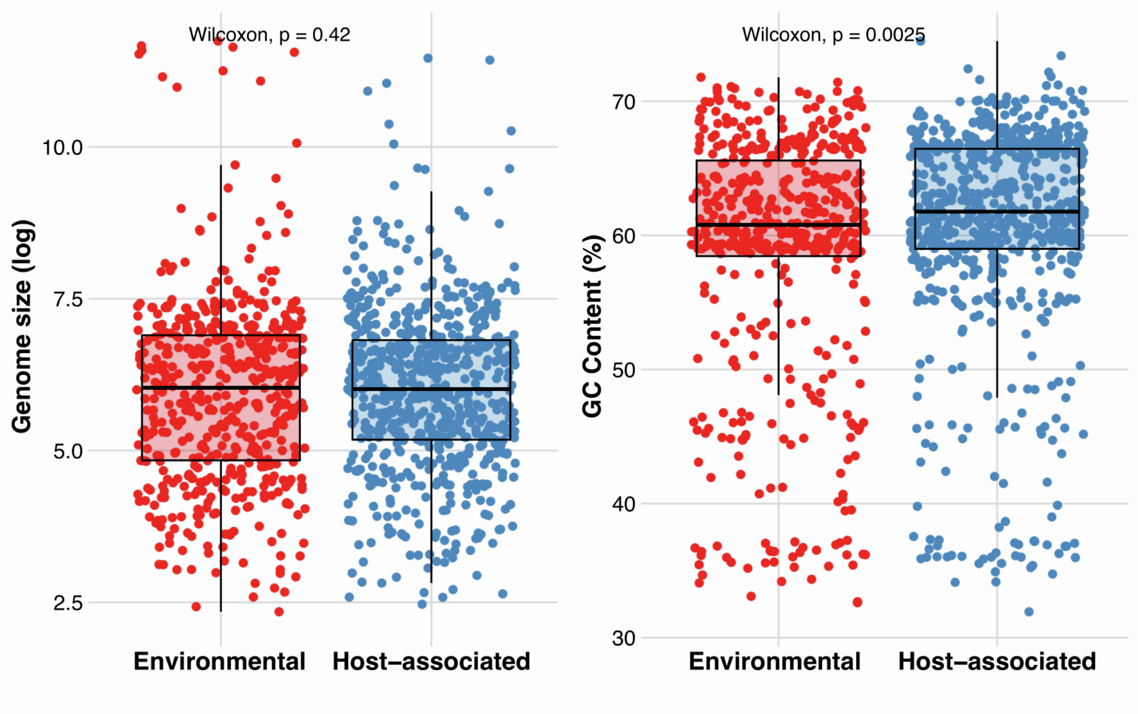

Figure S2: The distribution of genome sizes and GC content in environmental (soil, aquatic) and host-associated (Fungi, Human, Plants) bacterial genomes.

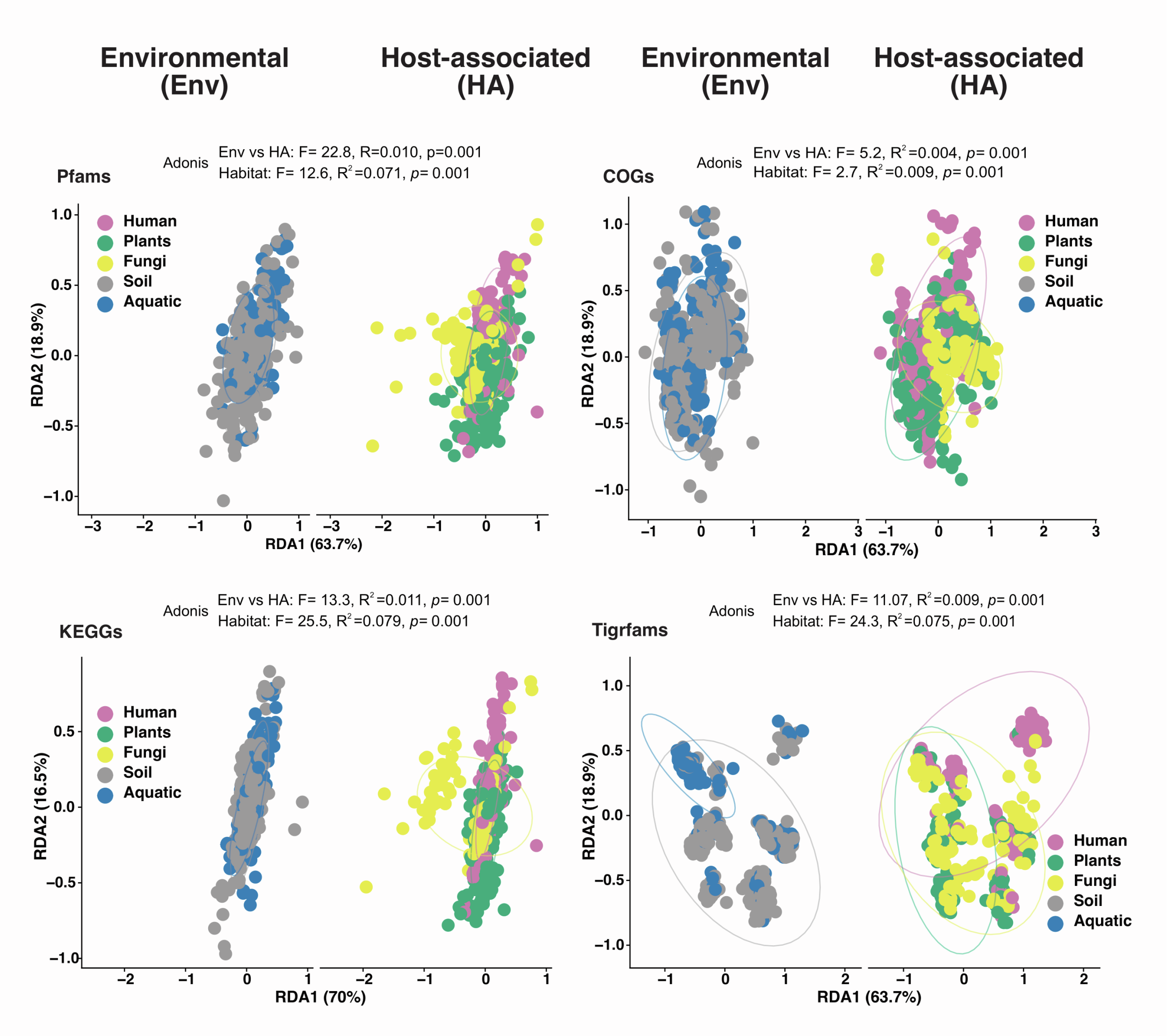

Figure S3: Cross-habitat distribution of genomic functional compositions based on different databases. RDA was performed based on Bray-Curtis distance dissimilarities.

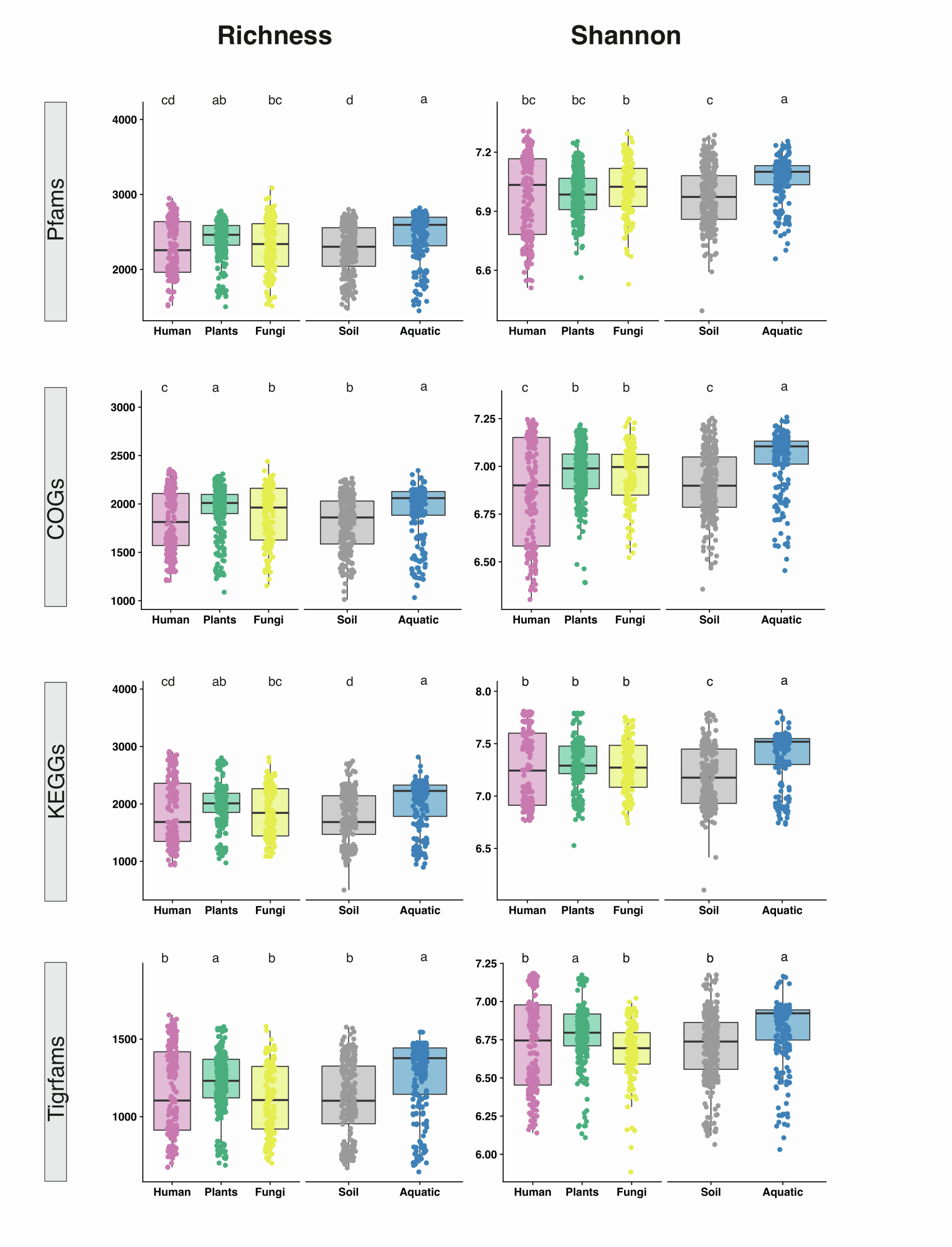

Figure S4: Diversity and richness of functional repertoires across all genomes and their respective origin. Pairwise comparisons were performed using ANOVA followed by post-hoc test (Tukey HSD). Different letters above each boxplot indicate significant differences between groups. Similar letters show similarity between the compared groups, whereas different letters denote a statistically significant difference.

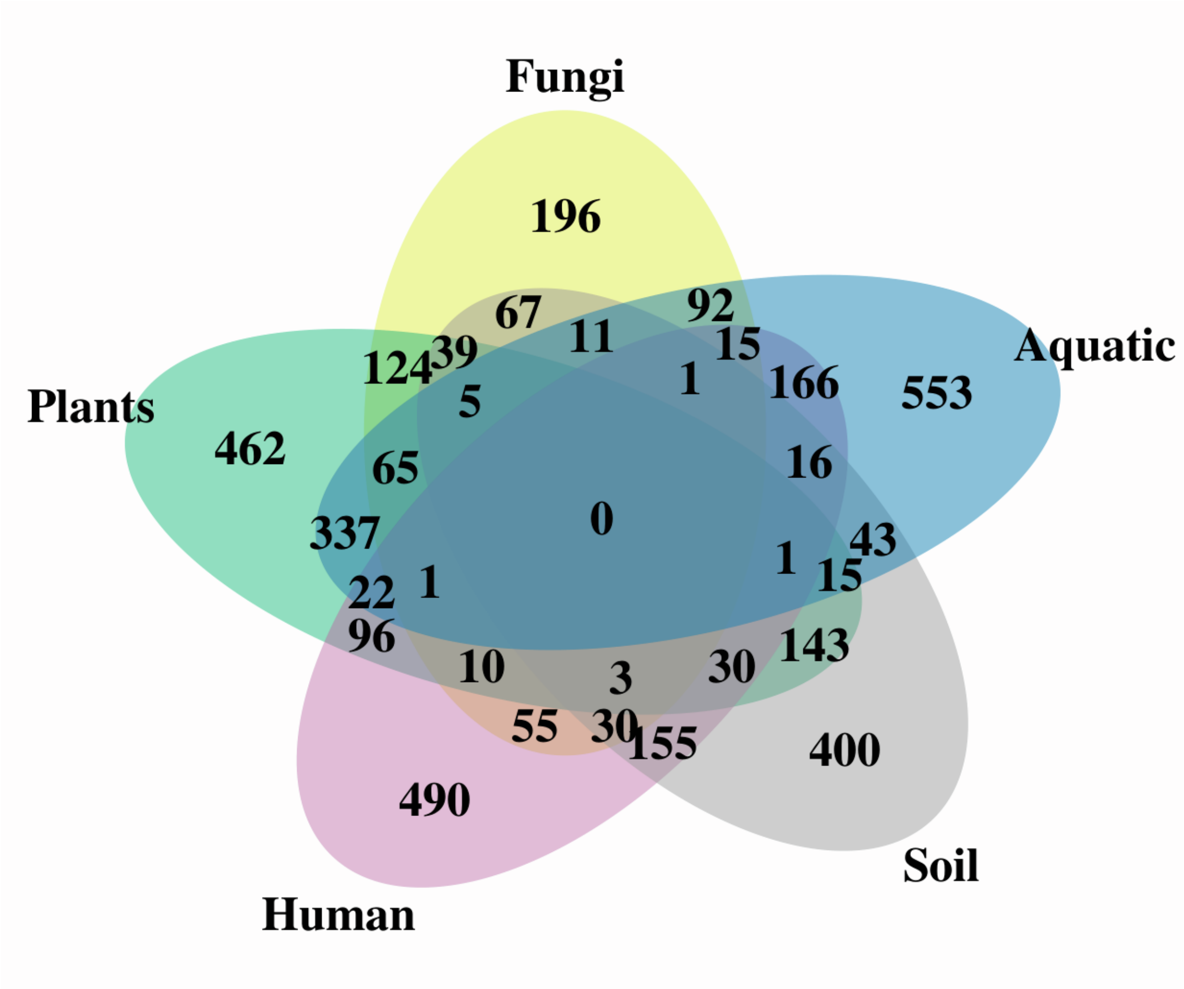

Figure S5: A Venn diagram depicting the distribution of habitat-associated genes based on hypergeometric tests, illustrating both the overlap and specificity of gene sets across multiple habitats.

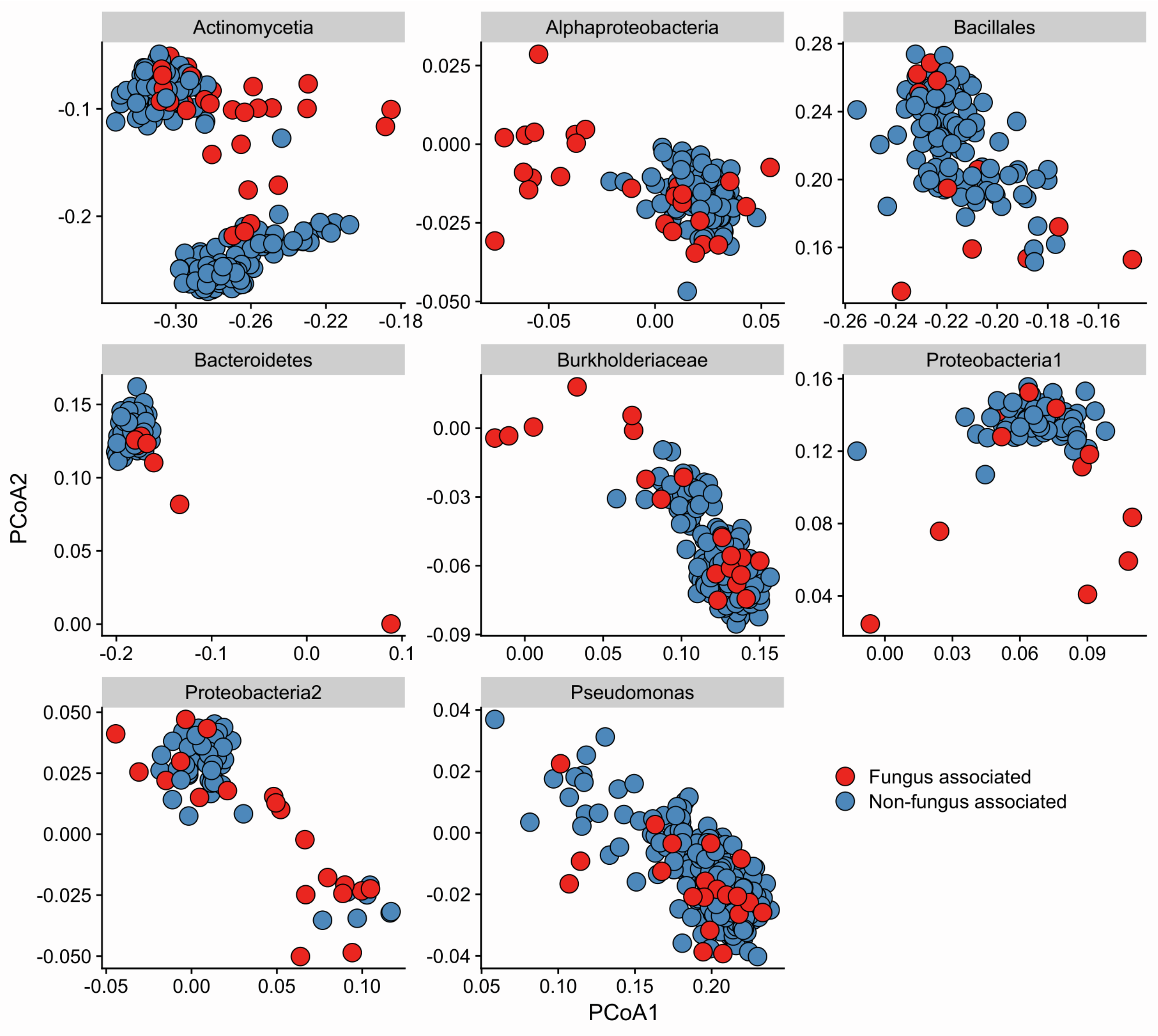

Figure S6: PCoA analysis based on matrices containing exclusively habitat-associated genes, obtained through hypergeometric tests. Only significant associations (FDR corrected p value<0.05) were considered as habitat-associated genes. To highlight the differences between FaB genes and those associated with other habitats, we merged the genes from other habitats and presenting them as a distinct group (non-FaB). Several taxa, including Alphaproteobacteria, Actinomycetia, Proteobacteria, Burkholderaceae, and Proteobacteria 1, show distinct clusters of fungus-associated and non-fungus-associated groups.

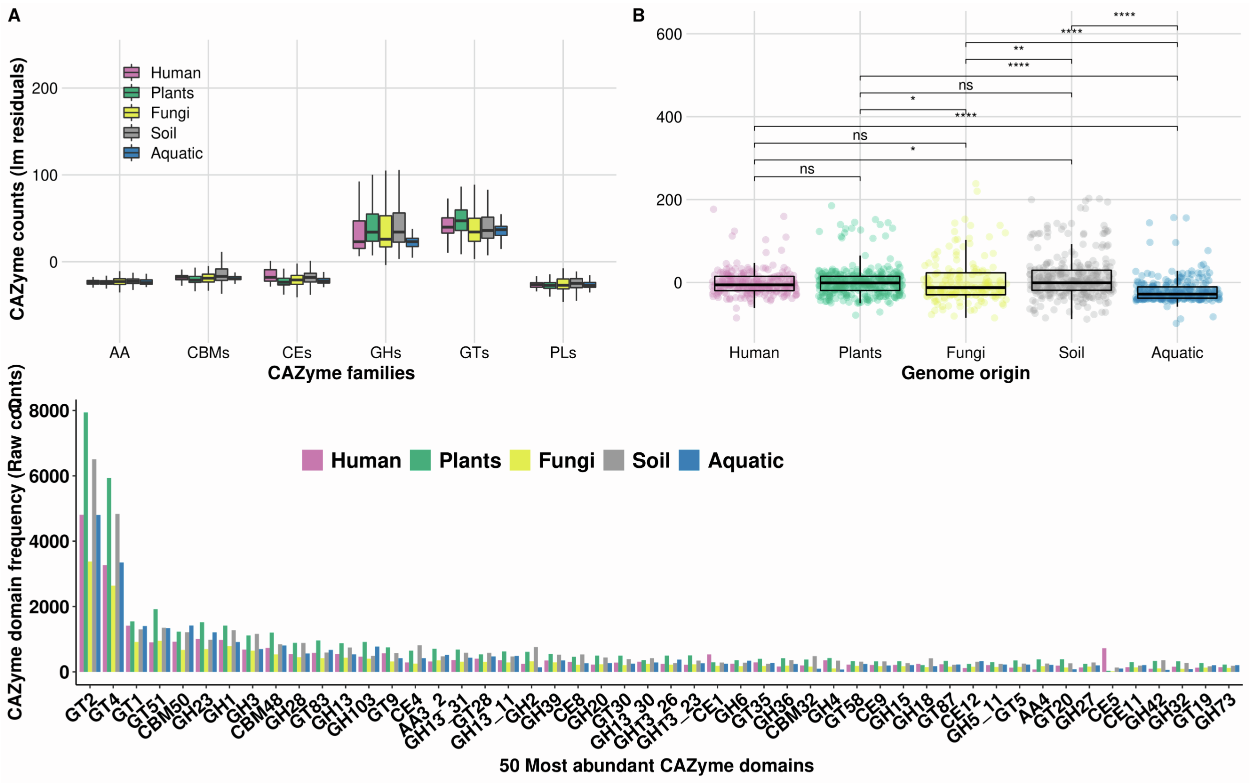

Figure S7: Distribution of CAZymes across bacterial genomes with their respective habitats. (A) Boxplot of CAZyme classes representing genomes from each habitat. The abundance of CAZyme from each genome was adjusted to their genome size using a linear regression model (lm). The Y-axis represents the residuals of lm. (B) comparison of overall abundance (lm residuals) of CAZymes across different habitats. Wilcoxon test significance and non-significance between pairs are shown with asterisks and ns, respectively. (C) 50 most abundant (raw counts) CAZyme families/domains across all the genomes studied.

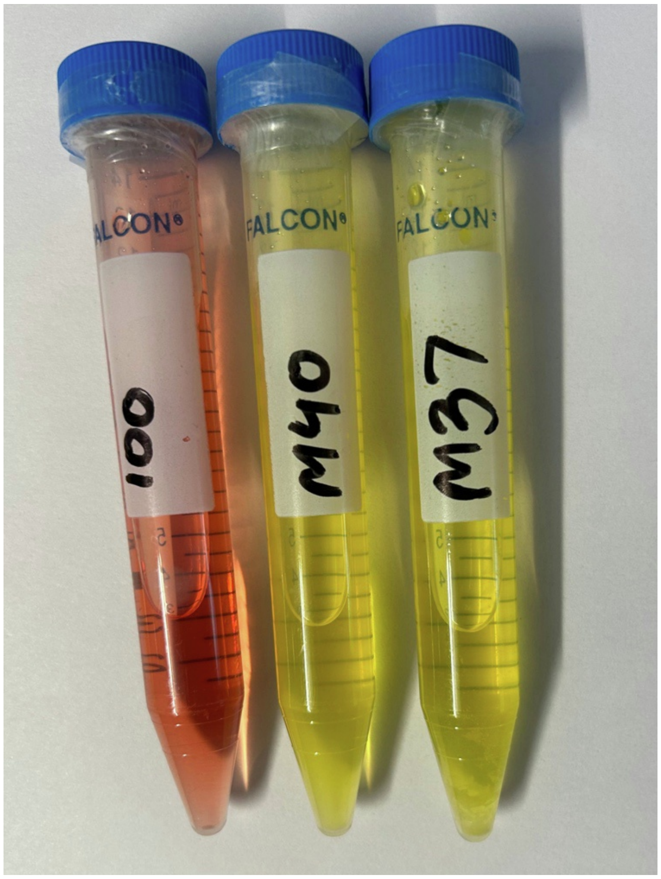

Figure S8: Ability of FaB isolates to degrade sucrose. We selected three isolates (*Pseudomonas* sp. 100, *Serratia fonticola* M40, *Enterobacteriaceae* sp. M37_B2 as referenced M37) based on their known possession of sucrose degrading enzymes. The inoculum from a pure culture was transferred aseptically to a sterile phenol red sucrose broth tube, followed by incubation at 35-37°C for 24 hours. The results were determined based on a color change from red to yellow, indicating a pH change to acidic. We observed that two of the isolates were able to degrade sucrose, as evidenced by the yellow color of the medium. This positive result suggests that these isolates are capable of producing acid end products from sucrose fermentation, leading to a decrease in pH and a subsequent color change in the medium
